# Winds of change: Hope for cleft lip and palate patients

**DOI:** 10.4103/0970-0358.57177

**Published:** 2009-10

**Authors:** Surajit Bhattacharya

**Affiliations:** Editor, IJPS, Sr. Consultant Plastic Surgery, Sahara Hospital, Lucknow, India. E-mail: surajitbh@yahoo.co.in

If patient satisfaction is a metre to calibrate specialists then the obstetrician surely scores over all others, as the overjoyed patient and her family are gifted every time with a bundle of joy. Second in this list will be the ophthalmologist as he/she transforms the dull and opaque world of the patient into a bright and colourful one. Third in this list surely is a plastic surgeon treating cleft lip and palate (CLP) patients. A 45-minute surgery transports a child from despair to delight; and anxiety to ecstasy. From being an object of curiosity, the child gets seamlessly assimilated and accepted in his play group. In short, it is a transformation from a life of ridicule to a life of hope and happiness ever after.

India, undoubtedly, has a very large cleft population, which at over a million is well above the entire population of some countries! Every passing year adds another 32,000 - 35,000 to the figure. Therefore, prevention should be our goal. The omnipresence of the sonologist in the far reaches of our population has aided in early detection of CLP in the unborn foetuses, offering parents a choice in medical termination of pregnancy. Though the foetuses are not in jeopardy of life or limb, this choice is socially accepted in India, though some human rights activists term this practice of “cosmetic murder” as the extremes of eugenics. Joanna Jepson, a British clergywoman, herself afflicted with a congenital jaw deformity, has initiated a legal suit to stem this practice in the UK.

Even as the genetic answer to this malady eludes humankind, the big challenge for research on cleft lip and palate is to make a quantum shift from studying individual genes to defining individual protein networks, a nexus of proteins that interact in a highly regulated manner. Since clefting is usually a sum of the combined actions of multiple gene products, it is likely that what we now call non-syndromic may have a distinct heterogeneous character, other manifestations being too subtle to manifest. Further research, however, might begin to better define these conditions.

The salutary role of Government Medical Colleges and non-governmental Organizations like the Smile Train in addressing the vast backlog of patients waiting for cleft surgeries can not be overemphasized. Due to their untiring efforts, though poised on the threshold of clearing this ‘quantity challenge’ of ever-growing numbers, it is time for us to switch gears and turn our attention to ‘quality’. Only an infinitesimal proportion of our operated patients get the benefit of orthodontics and speech pathology and for the majority, orthognathic surgery is a distant dream. Many of the top paediatric hospitals are developing their own cleft clinics in order to provide patients with comprehensive multi-disciplinary care from birth through adolescence.

Allowing an entire team to care for a child throughout their cleft lip and palate treatment allows for the best outcomes in every aspect of a child's care. While the individual approach can yield significant results, current trends indicate that team-based care leads to better outcomes for CLP patients. However, in a heterogenous India, we shall mostly continue to rely upon individual surgeons; and they shall harmoniously coexist with burgeoning multi-specialty centres of excellence.

There is a large amount of research dedicated to the psychosocial development of individuals with cleft palate. A CLP may impact an individual's self-esteem, social skills, and behavior; and they tend to report feelings of anger, sadness, fear, and alienation from their peers. Yet these children are similar to their peers with regard to “how well they liked themselves.”

Our Guest Editor, Dr. Jyotsna Murthy, has invited a galaxy of professionals working in the field of CLP to contribute to this issue and one can only marvel at both the quality and the quantity of research and clinical work under way, in all six continents, to bring back smiles to these thousands of afflicted faces.

